# Serum Uric Acid is Independently Associated with Enlarged Perivascular Spaces

**DOI:** 10.1038/s41598-017-16715-5

**Published:** 2017-11-27

**Authors:** Shuna Yang, Xiaoyu Zhang, Junliang Yuan, Jiangmei Yin, Wenli Hu

**Affiliations:** 1 Department of Neurology, Beijing Chaoyang Hospital Affiliated to Capital Medical University, Beijing, China; 2 Department of Neurology, Qianfoshan Hospital Affiliated to Shandong University, Jinan, Shandong Province China

## Abstract

Enlarged perivascular spaces (EPVS) are reported to be associated with impaired cognitive function and sleep disorders. It is of clinical importance to understand the risk factors for EPVS. Hyperuricemia increases the risk of hypertension and endothelial dysfunction, which are well recognized to be associated with EPVS. Therefore, we postulated that serum uric acid (SUA) might be associated with EPVS. A total of 665 lacunar stroke patients were enrolled in this study. The SUA concentrations of patients with severe EPVS were much higher than those of patients with mild EPVS (for basal ganglia: 5.25 ± 1.40 mg/dl vs. 4.75 ± 1.40 mg/dl, p < 0.001; for white matter: 5.31 ± 1.41 mg/dl vs. 4.88 ± 1.37 mg/dl, p = 0.009). The percentage of subjects with severe EPVS tended to be higher in the highest quartile of SUA (chi-square test: P = 0.002 for basal ganglia and 0.006 for white matter). Spearman correlation analysis indicated that the SUA concentrations were positively correlated with the severity of EPVS (rho > 0, p < 0.05). Multivariate logistic regression analysis showed that high normal SUA was independently associated with a higher severity of EPVS. This finding suggests that high SUA levels might be an independent risk factor for EPVS in lacunar stroke patients.

## Introduction

Enlarged perivascular spaces (EPVS), or Virchow-Robin spaces, appear as punctate or linear identical signal intensities that are similar to cerebrospinal fluid (CSF) on all MRI sequences in the white matter, basal ganglia and hippocampus^1,2^. EPVS are perivascular compartments that surround the small penetrating cerebral vessels, serving as an important drainage system for interstitial fluid and solutes in the brain^3^. It has been identified that EPVS are a marker of cerebral small vessel diseases^4,5^. Several studies have demonstrated that EPVS were associated with impaired cognitive function^1^, incident dementia^6^, depression, and sleep disorders^7^. Therefore, it is of clinical importance to understand the risk factors for EPVS and to search for future treatment options.

Uric acid (UA) is the end metabolic product of purine nucleotides. It has been reported that increased UA concentrations are associated with endothelial dysfunction^8^, hypertension^9–11^, diabetes^9,12^, and elevated serum triglyceride and cholesterol concentrations^13^, which increase the risk of EPVS. Therefore, we postulated that higher serum uric acid (SUA) concentrations might be associated with EPVS. However, some studies have suggested that UA can exert neuroprotective effects by acting as a free radical scavenger^14^. Higher level of SUA predicts better outcomes following stroke^15,16^. Administration of UA reduced ischemic damage and improved outcomes in an experimental model of stroke^17^. It is apparent that the function of UA remains controversial. An insufficient amount of research has been conducted to investigate the relationship between SUA and EPVS. In the present study, we aimed to explore whether SUA level is associated with EPVS.

## Results

### Baseline characteristics of the study participants

A total of 665 eligible participants with a mean age of 63 years old were enrolled in this study. None of the participants took uric acid lowering drugs or oestrogen within one week when their SUA concentrations were measured. The clinical and laboratory characteristics of all subjects and subgroups divided by the severity of EPVS are summarized in Table 1. Age, proportion of hypertension and Fazekas scale scores increased and HDL concentrations decreased with the degree of EPVS in basal ganglia increasing.

**Table 1:** The clinical and laboratory characteristics of all patients and subgroups stratified by the severity of EPVS.

Characteristics	Total sample	BG-EPVS			*F or chi-square values*	*P* value	WM-EPVS			*F or chi-square values*	*P* value
		mild	moderate	severe			mild	moderate	severe		
n	665	225	248	192	—	—	138	367	160	—	—
Age, years	63 ± 11	58 ± 10	62 ± 10	70 ± 10	74.75	<0.001	62 ± 11	63 ± 11	62 ± 11	1.67	0.189
Sex, male (%)	445(70%)	154(68.4%)	176(71.0%)	115(60.0%)	6.35	0.042	91(65.9%)	234(63.8%)	120(75%)	6.43	0.040
Smoking (%)	335(50%)	127(56.4%)	132(53.2%)	76(39.6%)	13.07	0.001	72(52.2%)	176(48.0%)	87(54.4%)	2.06	0.357
Alcohol (%)	227(34%)	86(38.2%)	96(38.7%)	45(23.4%)	13.75	0.001	44(31.9%)	113(30.8%)	70(43.8%)	8.72	0.013
Hypertension (%)	426(64%)	132(58.7%)	153(61.7%)	141(73.4%)	10.78	0.005	82(59.4%)	235(64.0%)	109(68.1%)	2.44	0.296
Diabetes (%)	209(31%)	72(32.0%)	79(31.9%)	58(30.2%)	0.19	0.910	42(30.4%)	124(33.8%)	43(26.9%)	2.55	0.280
CAD (%)	77(11.6%)	20(8.9%)	27(10.9%)	30(15.6%)	4.78	0.092	15(10.9%)	41(11.1%)	21(13.1%)	1.23	0.541
BMI, kg/m^2^	25.26 ± 3.21	25.11 ± 2.85	25.33 ± 3.31	25.35 ± 3.49	0.36	0.699	24.88 ± 3.15	25.25 ± 3.24	25.60 ± 3.19	1.86	0.156
HDL, mmol/L	1.26 ± 0.36	1.34 ± 0.34	1.25 ± 0.35	1.19 ± 0.37	8.94	<0.001	1.32 ± 0.34	1.26 ± 0.37	1.23 ± 0.34	2.34	0.098
LDL, mmol/L	2.69 ± 0.77	2.71 ± 0.74	2.68 ± 0.76	2.66 ± 0.83	0.19	0.830	2.63 ± 0.84	2.69 ± 0.74	2.73 ± 0.79	0.70	0.497
TC, mmol/L	4.74 ± 1.09	4.79 ± 1.02	4.70 ± 1.15	4.72 ± 1.10	0.43	0.650	4.74 ± 1.06	4.75 ± 1.13	4.71 ± 1.02	0.06	0.945
TG, mmol/L	1.77 ± 1.28	1.85 ± 1.46	1.73 ± 1.31	1.72 ± 1.00	0.75	0.475	1.86 ± 1.35	1.77 ± 1.34	1.68 ± 1.06	0.80	0.450
HbA1c, %	6.80 ± 1.92	6.65 ± 2.22	6.79 ± 1.74	6.98 ± 1.70	1.64	0.195	6.60 ± 1.72	6.87 ± 1.82	6.80 ± 2.24	1.02	0.361
Glu, mmol/L	6.69 ± 2.93	6.68 ± 3.38	6.58 ± 2.53	6.85 ± 2.85	0.45	0.635	6.55 ± 2.94	6.77 ± 3.04	6.64 ± 2.66	0.30	0.738
BUN, mmol/L	4.99 ± 2.80	4.91 ± 3.95	5.10 ± 2.22	4.94 ± 1.56	0.31	0.733	4.98 ± 2.67	5.12 ± 3.24	4.71 ± 1.49	1.20	0.302
Creatinine, umol/L	83 ± 28	81 ± 29	85 ± 31	83 ± 21	1.31	0.271	86 ± 36	82 ± 27	84 ± 22	0.80	0.449
SUA, mg/dl	4.98 ± 1.40	4.75 ± 1.40	4.98 ± 1.38	5.25 ± 1.40	6.77	0.001	4.88 ± 1.37	4.87 ± 1.39	5.31 ± 1.41	5.86	0.003
hs-CRP, mmol/L	3.05 ± 3.65	2.59 ± 2.94	3.35 ± 4.19	3.17 ± 3.61	2.73	0.066	3.13 ± 3.73	2.93 ± 3.56	3.23 ± 3.77	0.42	0.655
Fazekas scale	2.92 ± 1.72	1.88 ± 1.27	2.92 ± 1.63	4.13 ± 1.49	119.85	<0.001	2.43 ± 1.67	3.18 ± 1.74	2.72 ± 1.56	11.47	<0.001

BG, basal ganglia; WM, white matter; CAD, coronary artery atherosclerosis disease; BMI, body mass index; HDL, high-density lipoprotein; LDL, low-density lipoprotein; TC, total cholesterol; TG, total triglyceride; BUN, blood urea nitrogen; hs-CRP, hypersensitivity C response protein. Continuous variables are expressed as the mean values ± standard deviation and were compared with analysis of variance. Categorical variables were expressed as absolute numbers and percentages and were compared with chi-squared tests. Statistical significance was accepted at *p* < 0.05.

### Association between SUA Concentrations and EPVS

**Figure 1:**
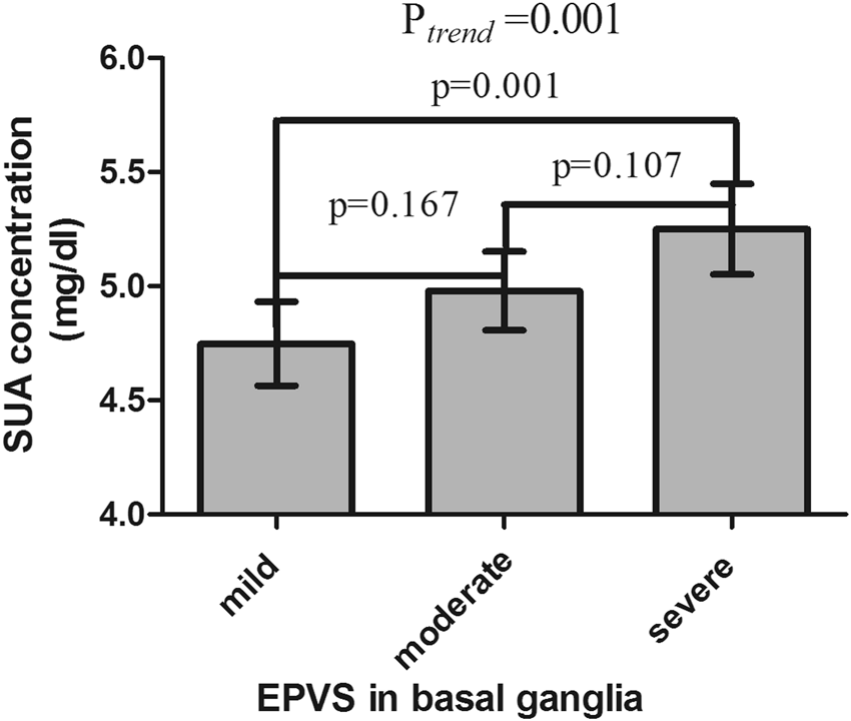
Histogram of the mean SUA concentrations in the subgroups divided by the severity of EPVS in basal ganglia. Analysis of variance was used among the three subgroups and Tukey tests were used in multiple comparisons. Statistical significance was accepted at *p* < 0.05. The histogram error bars indicate the 95% confidence interval (95%CI) of the SUA concentrations.

**Figure 2:**
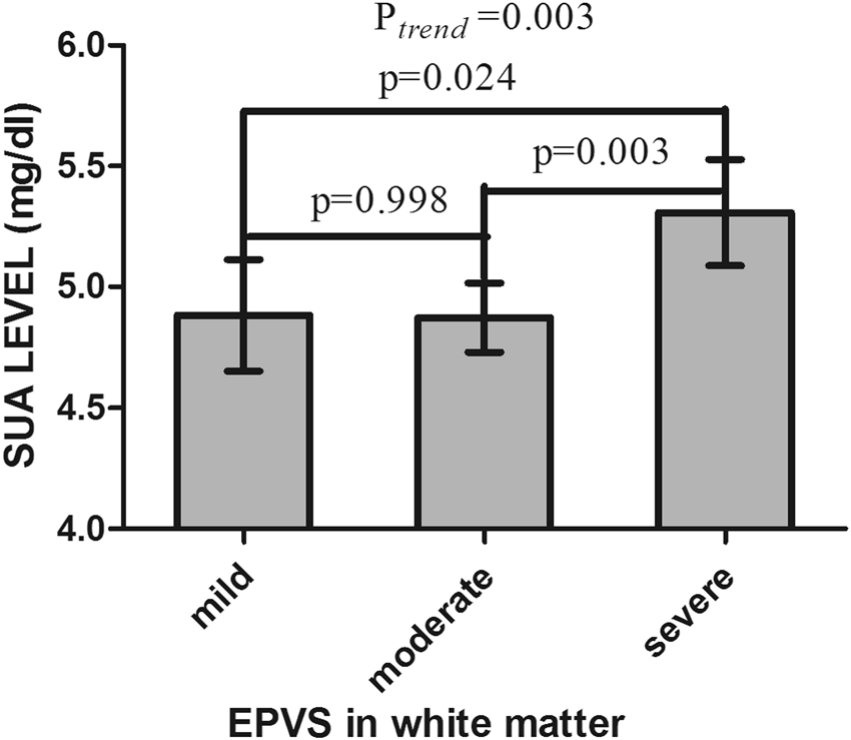
Histogram of the mean SUA concentrations in the subgroups divided by the severity of EPVS in white matter. Analysis of variance was used among the three subgroups and Tukey tests were used in multiple comparisons. Statistical significance was accepted at *p* < 0.05. The histogram error bars indicate the 95%CI of the SUA concentrations.

The SUA concentrations of each category divided by the severity of EPVS in different brain regions are presented in Table 1, Figs 1 and 2. As shown in Fig. 1, the SUA concentration increased with the severity of EPVS in basal ganglia (BG-EPVS) increasing. The results of Tukey tests showed that the SUA concentrations of patients with severe BG-EPVS were higher than those of patients with mild BG-EPVS (5.25 ± 1.40 mg/dl vs. 4.75 ± 1.40 mg/dl, p = 0.001). The SUA concentration of the severe BG-EPVS group was higher than that of the moderate group, and the concentration of the moderate group was higher than that of the mild group; however, there were no significant differences (5.25 ± 1.40 mg/dl vs. 4.98 ± 1.38 mg/dl, P = 0.107; 4.98 ± 1.38 mg/dl vs. 4.75 ± 1.40 mg/dl, P = 0.167). As shown in Fig. 2, the SUA concentrations of patients with severe EPVS in white matter (WM-EPVS) were also higher than those of patients with mild EPVS (5.31 ± 1.41 mg/dl vs. 4.88 ± 1.37 mg/dl, p = 0.024) and moderate EPVS (5.31 ± 1.41 mg/dl vs. 4.87 ± 1.39 mg/dl, p = 0.003). However, there was no difference in the SUA concentration when comparing between the mild EPVS group and moderate EPVS group (4.88 ± 1.37 vs. 4.87 ± 1.39, P = 0.998).

Spearman correlation analysis indicated that the SUA level was positively correlated with the severity of EPVS (for BG-EPVS: Spearman’s rho = 0.149, p < 0.001; for WM-EPVS: Spearman’s rho = 0.104, p = 0.007). However, the correlation was not strong. The results of multivariate logistic regression analysis are presented in Table 2. The odds ratio (OR) of comparing the severe BG-EPVS group to the mild BG-EPVS group was 1.436 (95% CI: 1.223–1.687) after controlling for demographic confounders (model 1) and 1.435 (95% CI: 1.210–1.701) after additionally adjusting for laboratory tests (model 2). The OR value increased to 1.466 (95% CI: 1.217–1.765) after additionally adjusting for Fazekas scale (model 3). Although there were negligible changes in statistical significance after adjusting for the Fazekas scale scores, the risk of the SUA concentration for the severe group did change. Individuals with severe BG-EPVS exhibited a 43.6% higher risk than those with mild BG-EPVS in model 1, and these results increased to 46.6% in model 3. After controlling for demographic confounders, laboratory tests and Fazekas scale scores, the SUA concentration was independently associated with the severity of WM-EPVS (model 1: OR = 1.214, 95% CI: 1.029–1.433; model 2: OR = 1.283, 95% CI: 1.084–1.519; model 3: OR = 1.270, 95% CI 1.074–1.501). However, the risk of the SUA concentration for severe WM-EPVS was lower than that for severe BG-EPVS. The statistical parameters of stepwise logistic regression analysis (forward method) were presented in Table 3, which showed the regression analysis models with statistical significance.

**Table 2:** Association between SUA and EPVS by multivariate logistic regression analysis.

	mild		moderate Odds ratio (95% CI)		severe Odds ratio (95% CI)	
	BG	WM	BG	WM	BG	WM
Model 1	Reference	Reference	1.182(1.031–1.355)	0.997(0.863–1.152)	1.436(1.223–1.687)	1.214(1.029–1.433)
Model 2	Reference	Reference	1.146(0.997–1.317)	0.989(0.856–1.143)	1.435(1.210–1.701)	1.283(1.084–1.519)
Model 3	Reference	Reference	1.134(0.982–1.310)	0.969(0.837–1.121)	1.466(1.217–1.765)	1.270(1.074–1.501)

Reference group: mild EPVS group.

Model1: adjusted for age and hypertension for BG-EPVS, adjusted for alcohol for WM-EPVS.

Model2: adjusted for age, hypertension and BUN for BG-EPVS,. adjusted for BUN for WM-EPVS.

Model3: adjusted for WMH severity, age, HDL, hypertension, BUN and HbA1c for BG-EPVS, adjusted for WMH severity and BUN for WM-EPVS.

**Table 3:** The statistical parameters of stepwise logistic regression analysis model.

Step	BG-EPVS				WM-EPVS			
	Effect(s) entry	P for effect selection tests	P for final model fitting	Goodness of fit (P_Pearson_)	Effect(s) entry	P for effect selection tests	P for final model fitting	Goodness of fit (P_Pearson_)
Model 1								
1	age	<0.001	<0.001	0.638	UA	0.003	0.001	0.441
2	UA	<0.001			alcohol	0.046		
3	HBP	0.008			—	—		
Model 2								
1	age	<0.001	<0.001	0.525	UA	0.003	0.001	0.493
2	HDL	<0.001			BUN	0.033		
3	UA	0.001			—	—		
4	HBP	0.009			—	—		
5	BUN	0.010			—	—		
Model 3								
1	Fazekas	<0.001	<0.001	0.436	Fazekas	<0.001	<0.001	0.447
2	age	<0.001			UA	0.002		
3	HDL	<0.001			BUN	0.047		
4	UA	0.004			—	—		
5	HBP	0.011			—	—		
6	BUN	0.009			—	—		
7	HbA1c	0.040			—	—		

The consequential significance level for entered and removal variables was 0.05 and 0.1, respectively.

Model1: Age, sex, smoking, alcohol, hypertension, diabetes, CAD and BMI were added.

Model2: model 1 + HDL, LDL, TC, TG, HbA1c, Glu, BUN were added.

Model3: model 2 + Fazekas scale score were added.

**Figure 3:**
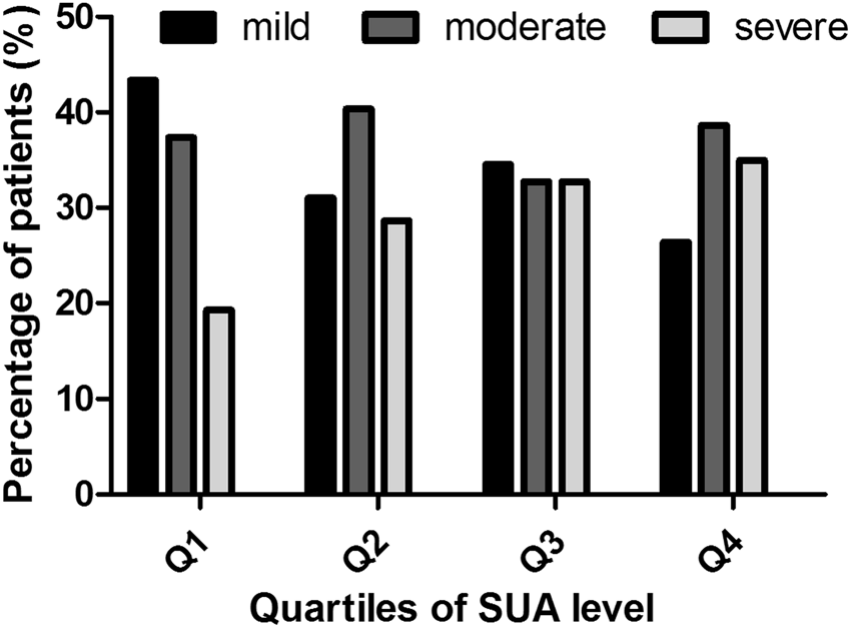
Distribution of the percentage of subjects in each quartile of SUA in different subgroups stratified by the severity of EPVS in basal ganglia. The percentage of subjects with severe EPVS tended to be higher in the highest quartile of SUA level.

**Figure 4:**
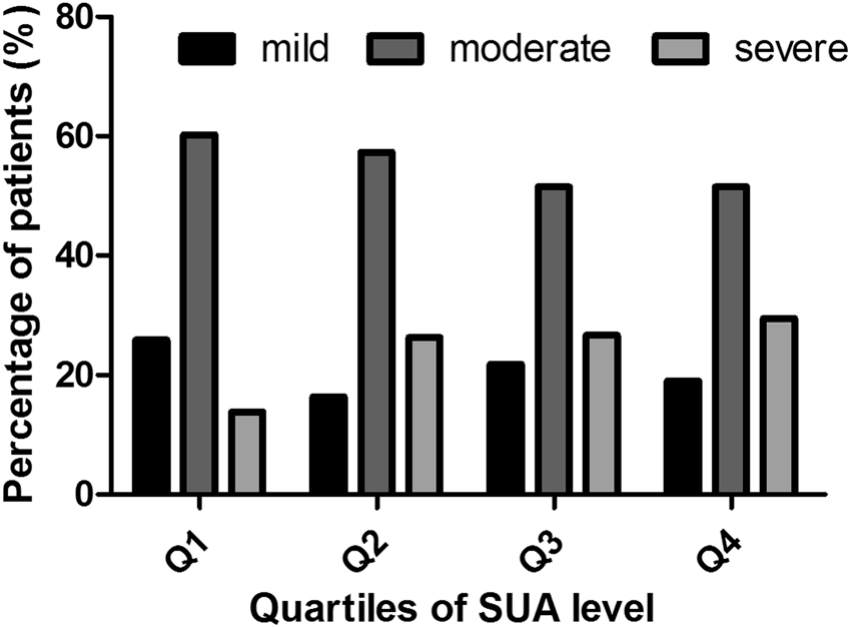
Distribution of the percentage of subjects in each quartile of SUA in different subgroups stratified by the severity of EPVS in white matter. The percentage of subjects with severe EPVS tended to be higher in the highest quartile of SUA level.

In addition, we further divided all subjects into quartiles by their SUA levels. Quartile 1 (Q1) was SUA ≤ 4.01 mg/dl, quartile 2 (Q2) was 4.01 mg/dl < SUA ≤ 4.91 mg/dl, quartile 3 (Q3) was 4.91 mg/dl < SUA ≤ 5.87 mg/dl, and quartile 4 (Q4) was SUA > 5.87 mg/dl. In the entire group of subjects, the percentages of subjects with severe EPVS tended to be higher in the highest quartile of the SUA level (chi-square test, P = 0.002 and 0.006, respectively) (Figs 3 and 4).

## Discussion

To our knowledge, this is the first study to investigate the relationship between the SUA concentration and EPVS. Our results revealed a positive correlation between SUA and the severity of EPVS in lacunar stroke patients. The SUA concentration increased when the degree of both BG-EPVS and WM-EPVS increased. The percentage of subjects with severe EPVS tended to be higher in the highest quartile of SUA. We found that a higher SUA concentration was an independent risk factor for EPVS by multivariable logistic regression analysis when adjusting for other confounders.

Many studies have explored the etiologies and potential pathophysiological mechanisms of EPVS. An increased permeability of the small vessel walls and blood brain barrier are considered to contribute to the development of EPVS, which has been reported to be associated with damage to microvascular endothelial cells and their tight junctions^3,18,19^. A large population-based study found that elevated interleukin-6 levels were associated with a higher severity of EPVS in the BG, suggesting that inflammatory responses may participate in the pathogenesis of EPVS^20^. In addition, leakage of interstitial fluid and structural changes of the microvascular wall may obstruct the drainage space and lead to consequent development of EPVS^21,22^.

Several mechanisms may be involved in the relationship between EPVS and SUA. One possibility is that high SUA concentration causes urate deposition in the small vessel walls, which directly damage the vascular endothelium and blood brain barrier. Another possible pathway is that UA induces endothelial dysfunction by reducing nitric oxide (NO) synthesis and availability^23,24^. In addition, high SUA concentration promote low-density lipoprotein oxidation *in vitro* ^25^ and stimulate granulocyte adherence to the endothelium^23,26^. Damage of microvascular endothelial cells and structural changes of the microvascular wall may result in leakage of interstitial fluid, obstruction of the drainage space and consequent development of EPVS^27^. Taken together, it is reasonable to conclude that high SUA concentration contributes to the development of EPVS through the combined effects of these factors.

The functions of UA are unclear mainly because of its many properties, including inducing oxidative stress, increasing inflammation, reducing NO synthesis and acting as a natural antioxidant^26,28^. Several studies have shown that low SUA concentration increases the risk of Alzheimer’s^29^ and Parkinson’s diseases^30^. In addition, animal experiments have indicated that administering UA reduces ischemic damage in rat models of acute stroke^17^. However, many studies have reported that elevated SUA concentration increases the risk of white matter hyperintensities (WMH)^31,32^, silent brain infarction^33^ and cardiovascular diseases^34^. Vannorsdall TD *et al*. found that older adults with high normal SUA concentration was more likely to perform poorly on cognitive measures^35^. Recent studies have demonstrated that EPVS are associated with impaired cognitive function^1,36^. In the present study, our results suggested that higher normal SUA concentration was associated with more severe EPVS, which was found to be significant even after controlling for confounding factors. Therefore, we speculated that EPVS might mediate the association between SUA and cognitive impairment, which should be demonstrated in future studies. Our results showed that the SUA concentration was within the normal range even in the severe EPVS group. Schretlen DJ *et al*. found that mild elevation of SUA (but within the normal range) might increase the risk of cognitive decline and brain ischemia^37,38^. We hypothesize that even a normal elevation of SUA may be associated with an increased burden of cerebral small vessel diseases, which might have broad public health implications. These findings might hold for the clinical values that respan the normal range of SUA.

There are some limitations in our study. First, our study population was based on lacunar stroke patients in a single centre; therefore, the cohort may not represent the general population. Second, although we found that higher SUA levels were correlated with a higher number of EPVS, the correlation was not strong. In addition, this was a cross-sectional study, and the causal relationship between SUA and EPVS could not be established. Further, our database did not include information on whether females were pre- or postmenopausal nor the dietary habits of the patients, such as the level of purine intake (for example meat and meat products).

In conclusion, our study indicated that elevated SUA concentration was positively correlated with the severity of EPVS. A clinical trial may be useful in determining whether reducing the production of SUA is beneficial in patients with cerebral small vessel diseases.

## Methods and Materials

### Ethics statement

All subjects provided written informed consent, and the study was approved by the Ethics Committee of Beijing Chaoyang Hospital affiliated to Capital Medical University and was performed in accordance with the Declaration of Helsinki.

### Study subjects and data collection

Consecutive small-artery occlusion (SAO) stroke patients defined by the Trial of Org 10172 in Acute Stroke Treatment (TOAST) classification^39^ were prospectively enrolled in the Neurology Department of Beijing Chaoyang Hospital affiliated to Capital Medical University from Jan 2011 to May 2015. Inclusion criteria were: (1) patients with an NIH Stroke Scale (NIHSS) score < 3; (2) patients undergoing brain MRI scans; (3) patients undergoing SUA measurements at least 7 days after SAO stroke onset; and (4) patients agreeing to participate in our study. The following patients were excluded: (1) patients with other stroke subtypes by the TOAST classification, Parkinson disease, dementia, severe traumatic or toxic or infectious brain injury, and brain tumour; (2) patients with severe heart disease and recent myocardial infarction or angina pectoris disorders, severe infections, severe nephrosis or liver disease, thrombotic diseases and tumour; and (3) patients unable to give written informed consent.

Clinical information including demographic data, past medical history, current smoking habits, alcohol consumption and medications were collected on all patients. Stroke severity was determined by the NIHSS score. All blood samples were collected in the morning after an overnight fasting period and were sent to the clinical laboratory of Beijing Chaoyang Hospital affiliated to Capital Medical University for the measurement of SUA concentrations. The SUA concentrations were measured by same method, using an autoanalyser in the clinical laboratory of our hospital.

### Assessments of EPVS and WMH

Neurological image examinations were performed on the same 3.0 T Siemens scanner (Erlangen, Germany) in the Radiology Department. Assessments of EPVS and WMH were performed by two experienced neurologists blinded to clinical information. Disagreements were resolved by consensus. The *k* statistic of the intra-rater and interrater agreement was 0.85 or above, indicating good reliability.

**Figure 5:**
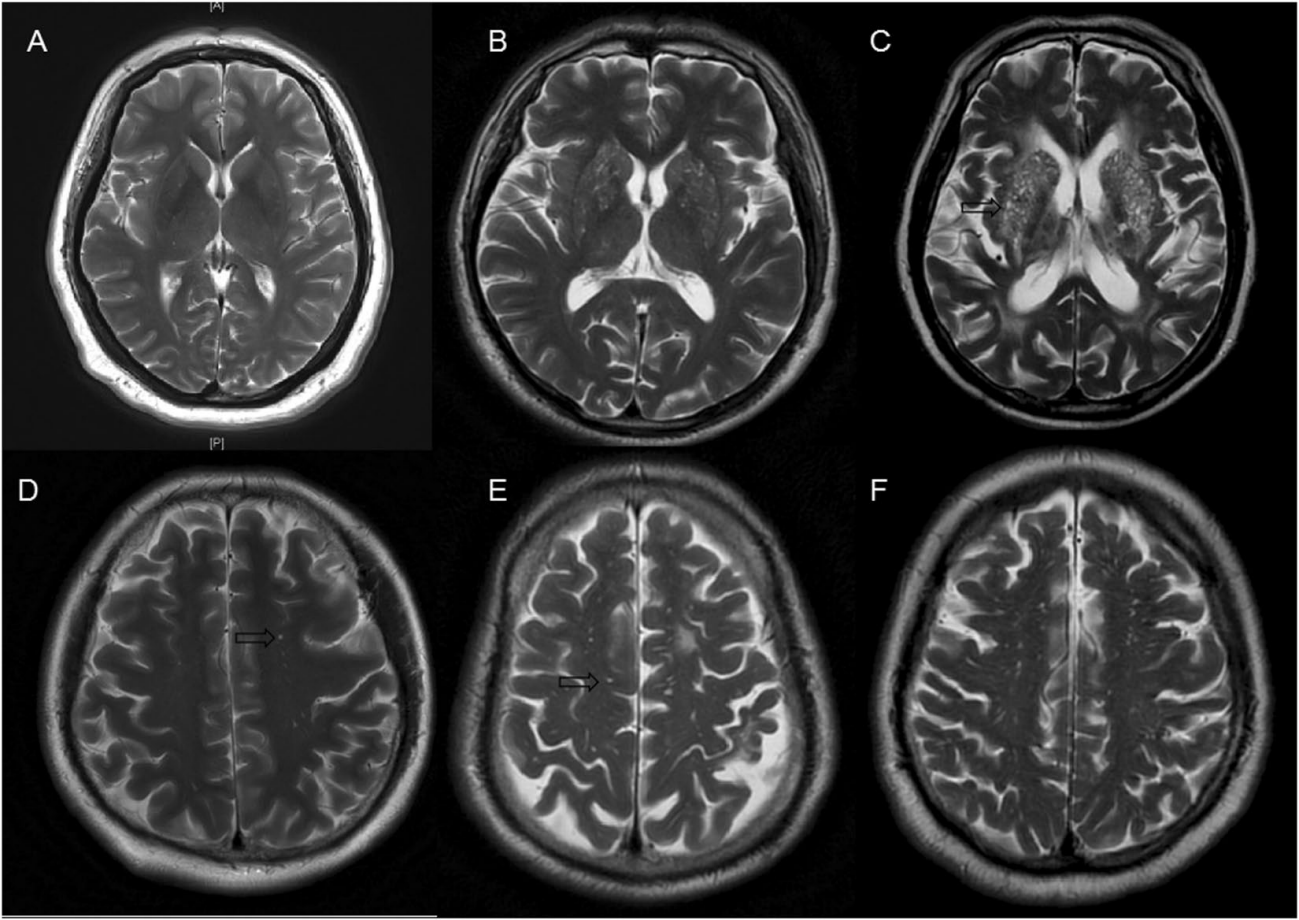
The severity of enlarged perivascular spaces in basal ganglia and white matter. (**A**) Mild in basal ganglia; (**B**) moderate in basal ganglia; (**C**), severe in basal ganglia; (**D**), mild in white matter; (**E**), moderate in white matter; (**F**), severe in white matter. The arrows were pointing to enlarged perivascular spaces, which appear as punctate or linear identical signal intensities that are similar to cerebrospinal fluid on MRI sequences.

EPVS were defined as CSF-like signal intensity lesions of round, ovoid, or linear shape of <3 mm and located in areas supplied by perforating arteries (Fig. 5). We distinguished lacune from EPVS by their larger size (>3 mm), spheroid shape and surrounding hyperintensities on FLAIR. BG-EPVS and WM-EPVS were separately assessed. The degree of EPVS severity was scored using a scale that was derived from previous studies: 0 = no EPVS, 1 =  ≤ 10 EPVS, 2 = 11 to 20 EPVS, 3 = 21 to 40 EPVS, and 4 =  > 40 EPVS^5,27^. The number referred to the highest number of EPVS on one side of the brain. We classified EPVS into three categories: mild = score 0 or 1; moderate = score 2; and severe = score 3 or 4. WMH were scored using the Fazekas scale. A detailed description of these assessments has been previously published^40^. Periventricular white matter hyperintensities (P-WMH) and deep white matter hyperintensities (D-WMH) were evaluated separately and summed as Fazekas scores.

### Statistical analysis

Continuous variables were presented as the mean values ± standard deviation and compared with analysis of variance (ANOVA) for factors with both normal distribution and homogeneity of variance. The Tukey test was used for multiple comparisons. Categorical variables were expressed as absolute numbers and percentages. The chi-squared test was used to test the independence of the categorical variables. Spearman correlation analysis was used to calculate the association between SUA levels and the severity of EPVS. Some vascular risk factors, such as age, sex, smoking, alcohol, hypertension, diabetes, serum lipid level, serum glucose level, renal function and the severity of WMH were reported to be associated with cerebral small vessel diseases. The multivariate logistic regression analysis was performed to assess whether SUA concentration was independently associated with EPVS after adjusting for these confounding factors. Given the high number of confounders considered, we adopted the stepwise logistic regression analysis model (forward method). The consequential significance level for entered and removal variables was 0.05 and 0.1, respectively. We also performed the correlation analysis among these variables. The correlation coefficient matrix indicated that the correlation were weak. Analysis was performed with Statistical Package for Social Sciences (SPSS version17.0), and statistical significance was accepted at *p* < 0.05.
